# Improving the identification of high-risk non-metastatic castration-resistant prostate cancer patients in clinical practice

**DOI:** 10.3389/fonc.2023.1266369

**Published:** 2024-01-23

**Authors:** Alina Rosinha, Carlos Rabaça, Fernando Calais, João Moreira Pinto, João Vasco Barreira, Ricardo Fernandes, Rodrigo Ramos, Ana Cristina Fialho, José Palma dos Reis

**Affiliations:** ^1^ Oncology Department, Portuguese Institute of Oncology (IPO) Porto, Porto, Portugal; ^2^ Urology Department, Portuguese Institute of Oncology (IPO) Coimbra, Coimbra, Portugal; ^3^ Urology Department, Centro Hospitalar Lisboa Central, Lisbon, Portugal; ^4^ Oncology Department, Hospital Beatriz Ângelo, Lisbon, Portugal; ^5^ Oncology Department, CUF Oncologia, Lisbon, Portugal; ^6^ Institute of Health Sciences, Universidade Católica Portuguesa, Lisbon, Portugal; ^7^ Oncology Department, Hospital de Braga, Braga, Portugal; ^8^ Urology Department, Portuguese Institute of Oncology (IPO) Lisboa, Lisbon, Portugal; ^9^ Medical Affairs Department, Janssen-Cilag, Lda., Porto Salvo, Portugal; ^10^ Urology Department, Hospital de Santa Maria, Centro Hospitalar Universitário Lisboa Norte, Lisbon, Portugal

**Keywords:** high-risk, identification, non-metastatic castration-resistant prostate cancer, prostate-specific antigen, prostate-specific antigen doubling time, standardization

## Abstract

Non-metastatic castration-resistant prostate cancer (nmCRPC) represents a challenging disease state in prostate cancer care. nmCRPC patients with a high risk of progression to metastatic disease who are identified by a prostate-specific antigen doubling time (PSADT) ≤10 months are eligible for treatment with the novel androgen receptor inhibitors (ARIs), shown to delay disease progression and extend survival. However, nmCRPC is often unexploited in clinical practice due to a lack of standardization in the methodology and in the tools used for its identification. In this article, a group of Urology and Oncology specialists with acknowledged expertise in prostate cancer reviews the state of the art in the management of high-risk nmCRPC patients, identifies gaps and unmet needs, and proposes strategies to optimize the identification of this patient subgroup in the clinical practice and improve their health outcomes.

## Introduction

According to recent data, prostate cancer ranked second as the most common cancer in men in 2020, accounting for an estimated 1.4 million diagnoses and 375,000 deaths globally that year (1). In Portugal, the latest data from the National Cancer Registry (RON) relates to 2019 and shows that prostate cancer was the most frequently diagnosed cancer in men that year, with 6912 new cases, and resulted in 1901 deaths (2), and GLOBOCAN 2020 reported 6759 new cases (20% of all cancers in men) and 1917 deaths from the disease (1).

Prostate cancer is a biologically heterogeneous entity, with histomorphologic and molecular characteristics that show substantial diversity between different patients (inter-patient heterogeneity) and within a given tumor (intra-tumoral heterogeneity), both in primary tumors (3–8) and in metastatic disease (9–12), with implications in diagnosis, clinical management, and prognosis (4, 13–15).

The clinical presentation can range from localized indolent to rapidly progressing metastatic disease (16–18). For patients presenting with early-stage disease at diagnosis, local therapy is often curative. However, more than 20% of patients experience biochemical recurrence, indicated by a rise in prostate-specific antigen (PSA) levels (19–21), and become eligible for first-line androgen deprivation therapy (ADT) (22). Progression during ADT, observed in 10−20% of patients (23), marks the onset of the castration-resistant state (24, 25), an advanced disease state with increased likelihood of metastases (26, 27). Around 15−30% of these patients become castration-resistant without imaging evidence of metastases, defining the non-metastatic (nm or M0) castration-resistant prostate cancer (CRPC) disease state (28–30).

The organ-specific serum marker PSA and its doubling time (PSADT), which measures the exponential increase in serum PSA over time (31), are used as measures of increasing cancer volume and prognostic biomarkers to guide treatment decisions. Guidelines recommend PSA testing every 3–4 months and imaging assessment every 3–6 months for patients with PSADT ≤ 10 months and every 6–12 months for those with PSADT >10 months (32). Despite these recommendations, PSA and imaging assessment are underused in the real-world practice after initiation of continuous ADT, as shown in a large, population-based cohort study where more than half of patients with CRPC who progressed to high-risk nmCRPC received ≤2 PSA tests in the previous year and 31% received no imaging studies in the following 12 months (33). Infrequent patient monitoring hinders proper disease staging, risk stratification, and detection of treatment failure and/or metastases, with suboptimal patient outcomes. Both the joint EAU-EANM-ESTRO-ESUR-SIOG and ESMO guidelines strongly recommend the use of PSADT to define the risk of recurrence in prostate cancer after radical prostatectomy and radiation therapy (22, 34).

PSADT represents the number of months it takes for PSA to increase two-fold and is calculated assuming an exponential rise in serum PSA. The formula takes into account the natural logarithm of 2 divided by the slope obtained from fitting a linear regression of the natural log of PSA over time, i.e., PSADT = [ln(2)*/IT]/[ln(PSA final) – ln(PSA initial)] (35). However, several different PSADT definitions have been used according to the mathematical formula employed and the PSA values included (number, time period, intervals) (36), with the values retrieved also shown to vary widely among calculations (37). For example, the Memorial Sloan Kettering Cancer Center method calculates a regression slope integrating all PSA values, while other methods do not include all PSA values and transform PSA before estimating the slope (37). Regardless of these disparities, according to the EAU - EANM - ESTRO - ESUR - ISUP - SIOG guidelines, some rules can be assumed for PSADT calculation (34):

- At least 3 PSA measurements are required (35);
- A minimum time period between measurements (4 weeks) is preferable due to potential statistical ‘noise’ when PSA values are obtained too close together (this can be reconsidered in case of very active disease);
- All PSA values should be >0.20 ng/mL and follow a global rising trend;
- All included PSA values should be obtained within the past 12 months at most, to reflect the current disease activity;
- PSADT is often mentioned in months, or in weeks in highly active disease.

## Non-metastatic castration-resistant prostate cancer − a challenging disease state

nmCRPC is a disease stage defined by a very specific diagnosis established within a sensitive time period. It is formally defined by a 25% rise in PSA levels from nadir (starting at ≥1.0 ng/mL and with a minimum rise of 2.0 ng/mL) in the presence of castrate testosterone levels (<50 ng/dL) and absence of detectable disease on conventional imaging (computed tomography [CT] and bone scan or magnetic resonance imaging [MRI]) (24, 25). This PSA rise must be confirmed by a second value obtained ≥3 weeks later in the same context of castrate testosterone levels (<50 ng/dL) (24).

Data about the prevalence of nmCRPC is scarce, with one study from 2013 estimating it to range from 2−8% in different countries, with a trend toward an increase over the next years due to widespread PSA screening (38).

Patients are mostly asymptomatic, with data retrieved from the placebo arms of clinical trials indicating that up to 60% develop overt metastatic disease within 3−5 years (29, 39–41). However, these patients were assessed with conventional imaging methods (CT/MRI), and more recent evidence suggests that some may already have metastatic disease if prostate-specific membrane antigen ligand positron emission tomography (PSMA-PET) imaging had been employed instead. Indeed, emerging studies suggest that PSMA-PET detects prostate cancer with superior sensitivity to conventional imaging, as the study by Fendler et al., in which PSMA-PET was positive in 196 of 200 patients with high-risk nmCRPC and detected 55% of M1 disease and 44% of pelvic disease despite negative conventional imaging (42); and the study by Fourquet and colleagues, which used PSMA-PET to restage 30 nmCRPC patients and found at least one malignant focus in 90% of those (43). Notwithstanding, the performance of PSMA-PET in nmCRPC is still poorly studied and there are no phase III trials showing a survival benefit with its use versus CT/MRI to guide treatment decisions. This is also reflected in the recent EAU-EANM-ESTRO-ESUR-ISUP-SIOG guidelines, which do not consider PSMA-PET in the management of nmCRPC (34). Overall, the use of PSMA-PET in nmCRPC at this point is still being investigated.

Several prognostic markers of metastasis-free survival (MFS) and overall survival (OS) have been identified in nmCRPC, including baseline PSA (29, 40), PSA velocity (29), PSA at CRPC diagnosis (44, 45), and PSADT (39, 46). Since the presence of metastatic disease is associated with increased morbidity and mortality and decreased quality of life (47), preventing or delaying progression to metastatic state is the primary therapeutic goal in the nmCRPC patient population (47).

Until 2018, there was no standard of care for patients with nmCRPC progressing on ADT, neither PSA or PSADT cut-offs to guide treatment decisions. Patients were usually managed with a watchful waiting approach until the detection of metastases or with loco-regional treatments (48). Maximal androgen blockade, through the addition of a first-generation antiandrogen (e.g., bicalutamide) to ADT, and switching or withdrawal of antiandrogens were sometimes offered to these patients without evidence of a survival benefit demonstrated in clinical trials, with short-term PSA responses and limited benefit (49–52).

The introduction of next-generation androgen receptor inhibitors (ARIs) apalutamide, darolutamide, and enzalutamide changed the therapeutic landscape of prostate cancer, providing therapy options with improved outcomes, including for nmCRPC patients. These agents have been shown to extend MFS in nmCRPC patients with no detectable metastases on conventional imaging and a PSADT <10 months on continuous ADT (i.e., at high risk of developing metastatic disease) when added to ADT in the respective landmark phase 3 SPARTAN, ARAMIS, and PROSPER trials (53–55). In subsequent analyses with longer follow-up, they also demonstrated a survival benefit for these patients, reducing the risk of death compared to placebo by 22–31% (56–58). These results led to the recommendation in international guidelines for the use of apalutamide, darolutamide, or enzalutamide in addition to ADT in patients with nmCRPC and a PSADT <10 months (34, 59), with factors like treatment toxicity, patient comorbidities, drug interactions, and access determining the choice of the best agent for each individual patient.

## Timely identification of nmCRPC patients with high risk of metastatic disease – an unmet need

PSA and PSADT are prognostic biomarkers used to guide therapy intensification with life-prolonging therapies in nmCRPC. Both have been linked to patient outcomes in this setting, with patients who present a rise in PSA level and a short PSADT (PSADT ≤10 months) bearing a higher risk of metastatic progression and death (29, 39, 40, 60).

Regular PSA monitoring and imaging assessment are crucial for identifying nmCRPC prior to the development of metastases, particularly given that these patients are often asymptomatic (61, 62). In addition, regular PSA monitoring allows the accurate calculation of PSADT, crucial for prognostic risk assessment in nmCRPC and for making evidence-based decisions toward therapy intensification through the addition of ARIs to the treatment backbone, delaying the development of metastases and improving survival (53, 54, 58). In a recent retrospective study, among 944 nmCRPC patients, 97.6% progressed to high-risk disease with PSADT ≤10 months (33), highlighting the relevance of this biomarker in the identification of the high-risk nmCRPC state.

Although the treatment landscape for nmCRPC has substantially evolved, the timely identification of high-risk nmCRPC patients in the clinical practice remains an unmet need. This is mainly due to inconsistency in the frequency of PSA monitoring in the routine practice and therefore in the calculation of PSADT, and to a lack of standardization in the methodology and tools used to do this calculation. These patients are mainly followed in Urology and Oncology setting, where the clinical practice of identification of the high-risk state has been variable and non-systematic, both regarding the frequency of PSADT assessment and the tools used to do it. And this represents a significant barrier for successfully incorporating the currently available treatments into the real-world practice and offering these patients a more favorable prognosis.

It is therefore crucial that urologists and oncologists managing nmCRPC patients adopt and routinely apply proper tools to calculate PSADT and make treatment decisions for their patients accordingly. Some tools are already available to allow healthcare providers to accurately estimate PSADT at the point of care and assist them in their decision-making process. These include the online PSADT calculator and two materials that resulted from the conversion into physical format of the results retrieved by the online calculator and have the potential to be more easily accessed in the daily clinical practice and more convenient for the clinician: the PSA Do-IT ruler and the PSA Do-IT table.

### Online PSADT calculator

The PSADT calculator (https://www.mdapp.co/psa-doubling-time-calculator-535/) is an online tool for determining the number of months it takes for PSA levels to double. The scientific rationale and calculations for the development of the calculator are based on publications in the literature (36, 63) and are detailed on the calculator’s website. The accuracy of the estimate improves as more PSA values are entered into the calculator. Therefore, for prostate cancer patients with biochemical recurrence, an optimal PSADT calculation should include as many of the patient’s PSA values as possible within two years of the first documented PSA recurrence. A shorter PSADT (≤6 months) is a negative predictor, reflecting a faster increase in PSA levels, while a longer PSADT (>6 months) is a positive predictor, reflecting a slower increase in PSA levels over time (63–66).

### PSA Do-IT ruler

**Figure 1:**
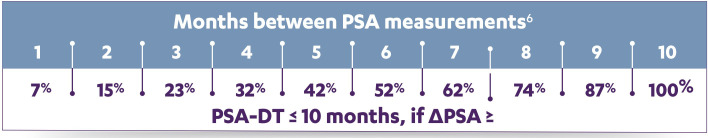
PSA Do-IT ruler for identifying patients with PSADT ≤10 months in the clinical practice.

The PSA Do-IT ruler is a material resource developed by Janssen Portugal directly derived from the online PSADT calculator to quickly and easily identify patients with a PSADT ≤10 months in clinical practice (**Figure 1**). It can be carried in the pocket or kept on the office desk and used to quickly screen and identify these patients based on the months between PSA measurements and the percentage PSA variation, allowing a quick assessment of the patient’s risk status.

### PSA Do-IT table

**Figure 2:**
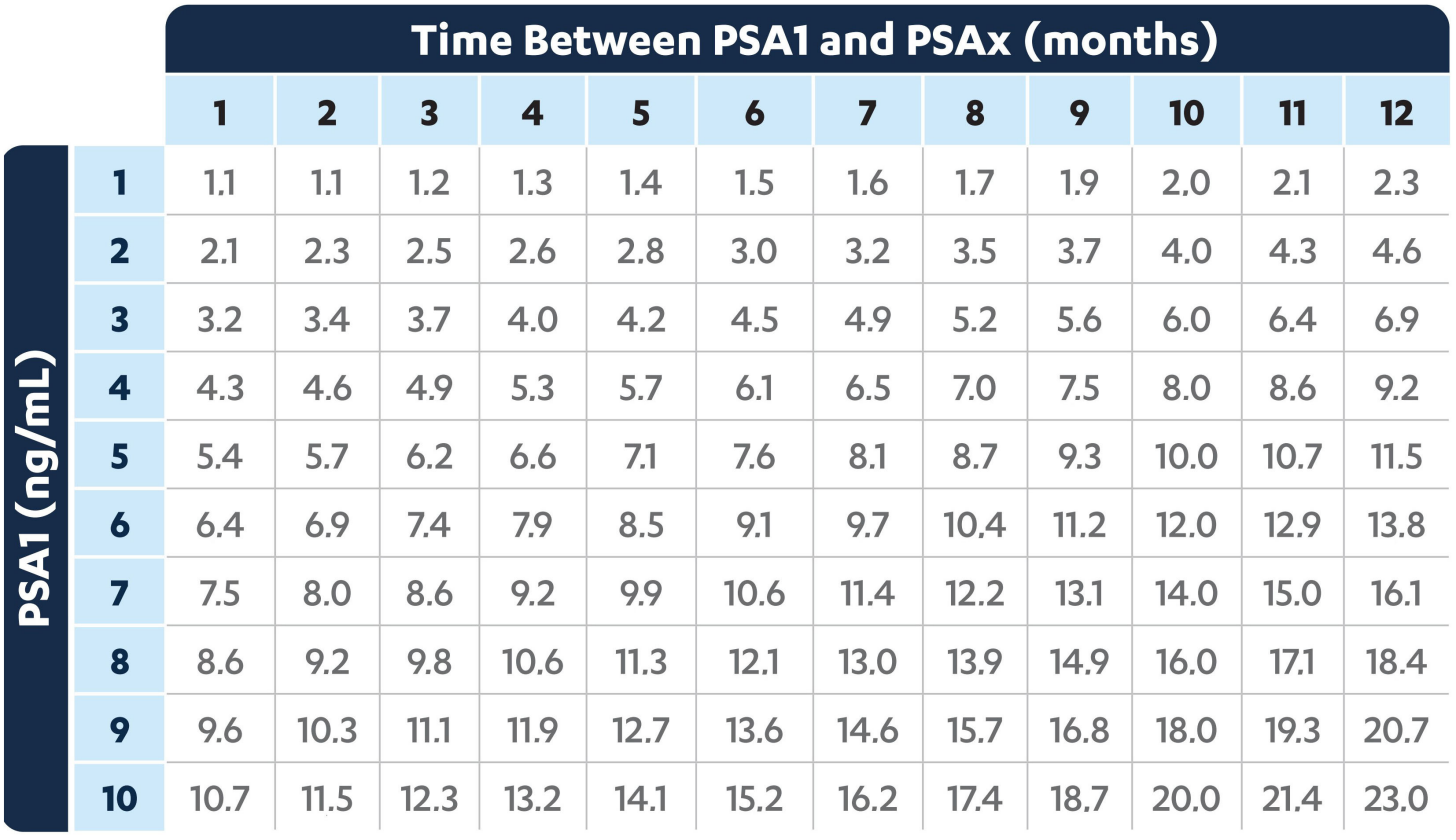
PSA Do-IT table for identifying patients with PSADT ≤10 months in the clinical practice.

The PSA Do-IT table is another material resource developed by Janssen Portugal to quickly identify patients with a PSADT ≤10 months, based on the results obtained by the online PSADT calculator (**Figure 2**). By combining two different PSA levels and the time between measurements, the table provides a reference PSA value. Increases in PSA above those shown in the table identify patients with a PSA-DT ≤ 10 months.

## Discussion

The identification of high-risk nmCRPC patients is an evidence-based process that relies on an objective assessment: a PSADT value ≤10 months. For it to be effective, this assessment should be done regularly and using a standardized approach, but it is currently hindered by several difficulties in the daily practice and by a lack of standardization in the methodology used to do it.

Total PSA assessment and PSADT calculation are the methods preferred by specialists treating prostate cancer for identifying high-risk nmCRPC patients. PSADT is a widely acknowledged and validated method for risk stratification and definition of eligibility for treatment with new-generation ARIs, as it was the method used in clinical trials of these agents and is foreseen in the guidelines. It is not the only risk stratification tool used, with Gleason Score also having a prominent role.

However, urologists and oncologists identify several obstacles in the clinical practice that hamper the management of nmCRPC patients and consequently the identification of the high-risk disease state. Among these are lengthy diagnostic procedures, difficulties in scheduling imaging exams in due time, difficulties in scheduling appointments and in following patients for PSA assessment in time, and short consultation time, among others. Due to these obstacles, PSA assessment and consequently PSADT calculation are done inconsistently and heterogeneously among specialists, clinical practices, and patients. Some physicians estimate PSADT in every patient visit, with the periodicity of visits depending on the previous PSADT value and/or PSA kinetics; others recalculate PSADT at each patient visit in the case of patients who are in castration resistance with evidence of biochemical progression; others calculate PSADT every 3 months, possibly extending the interval to every 6 months if PSADT is very low; some only calculate PSADT at the time of the multidisciplinary Urology consultation, after the patient has been diagnosed with nmCRPC in the Urology consultation and referred to multidisciplinary follow-up; others calculate PSADT on a patient-by-patient basis according to the patient’s PSA kinetics at least twice a year. This overall lack of consistency and standardization in the procedure for identifying high-risk patients, together with the narrow time window to do it and lack of awareness of some healthcare providers about its importance challenge the optimal management of this patient subgroup, as there is the risk of missing patients who could otherwise benefit from treatment with ARIs.

Among the tools currently available to identify high-risk nmCRPC patients, the PSA Do-IT Ruler and PSA Do-IT Table are considered good and useful materials to raise awareness for PSADT calculation, but the online PSADT calculator remains the preferred and most frequently used tool among physicians.

Given this scenario, the identification of high-risk nmCRPC patients can and should be optimized, allowing patients to achieve the best health outcomes. Incorporating the PSADT calculation into laboratory request forms with the remaining analyses has the potential to be a reminder of this assessment and facilitate and expedite PSADT calculation. Implementing a physician alert when the patient has a PSADT ≤10 months, with subsequent referral to multidisciplinary group meeting, and optimizing analytical procedures and hospital appointment scheduling are additional strategies that can be used to improve the identification of high-risk nmCRPC patients in the clinical practice.

Regardless of these measures, the importance of routine PSADT calculation should be reinforced among the medical community, and awareness should be raised to its relevance as a key step to offer patients the best treatment approach. This can be achieved through the development of training and awareness initiatives directed at medical specialists who follow these patients, as well as multidisciplinary meetings with specialties involved in their management.

According to the guidelines, once identified, nmCRPC patients with PSADT ≤10 months indicative of a high risk of metastases should be treated with ARIs in combination with ADT. However, this is not the only criterion, as individual patient characteristics also impact the treatment decision. Some patients with uncontrolled comorbidities, poor performance status, and/or reduced life expectancy may not be eligible for treatment with the novel ARIs and a watchful waiting approach may be more indicated. On the other hand, biological age is not an absolute exclusion criterion for treatment eligibility. Patient and family expectations should also be assessed and considered in the treatment decision, validating their understanding of the treatment clinical benefit. Overall, the experts consider that the focus should be on the patient and not on the disease.

## Conclusion

Overall, the patterns of care for patients with nmCRPC are under-optimized, in particular for those with high-risk disease. The current practice in the management of these patients is of under- and non-standardized monitoring, precluding timely institution of treatment with a direct impact on patients’ outcomes. The treatment landscape for high-risk nmCRPC has substantially evolved, but the routine and standardized calculation of PSADT in these patients’ clinical practice remains an unmet need, as only the confirmation of PSADT ≤10 months provides formal indication to treat patients with novel and effective therapies. There are several ways to optimize this process in the clinical practice, such as incorporating the PSADT calculation into the laboratory request form, creating physician alerts for patients with PSADT ≤10 months, with subsequent referral to multidisciplinary assessment, and optimizing analytical procedures and scheduling of appointments at the hospital. The successful implementation of these measures will predictably improve the real-world patterns of care for nmCRPC patients.

## Data availability statement

The original contributions presented in the study are included in the article/supplementary material. Further inquiries can be directed to the corresponding author.

## Author contributions

AR: Formal Analysis, Validation, Writing – review & editing. CR: Formal Analysis, Validation, Writing – review & editing. FC: Formal Analysis, Validation, Writing – review & editing. JMP: Formal Analysis, Validation, Writing – review & editing. JVB: Formal Analysis, Validation, Writing – review & editing. RF: Formal Analysis, Validation, Writing – review & editing. RR: Formal Analysis, Validation, Writing – review & editing. ACF: Conceptualization, Formal Analysis, Validation, Writing – review & editing. JPR: Formal Analysis, Validation, Writing – review & editing.
